# Near-Infrared Spectroscopy Enables Arthroscopic Histologic Grading of Human Knee Articular Cartilage

**DOI:** 10.1016/j.asmr.2022.07.002

**Published:** 2022-08-20

**Authors:** Jaakko K. Sarin, Mithilesh Prakash, Rubina Shaikh, Jari Torniainen, Antti Joukainen, Heikki Kröger, Isaac O. Afara, Juha Töyräs

**Affiliations:** a Department of Applied Physics, University of Eastern Finland, Kuopio, Finland; b Department of Medical Physics, Medical Imaging Center, Pirkanmaa Hospital District, Tampere, Finland; c A.I. Virtanen Institute for Molecular Sciences, University of Eastern Finland, Kuopio, Finland; d Department of Orthopedics, Traumatology and Hand Surgery, Kuopio University Hospital, Kuopio, Finland; e School of Information Technology and Electrical Engineering, The University of Queensland, Brisbane, Australia; f Science Service Center, Kuopio University Hospital, Kuopio, Finland

## Abstract

**Purpose:**

To develop the means to estimate cartilage histologic grades and proteoglycan content in ex vivo arthroscopy using near-infrared spectroscopy (NIRS).

**Methods:**

In this experimental study, arthroscopic NIR spectral measurements were performed on both knees of 9 human cadavers, followed by osteochondral block extraction and in vitro measurements: reacquisition of spectra and reference measurements (proteoglycan content, and three histologic scores). A hybrid model, combining principal component analysis and linear mixed-effects model (PCA-LME), was trained for each reference to investigate its relationship with in vitro NIR spectra. The performance of the PCA-LME model was validated with ex vivo spectra before and after the exclusion of outlying spectra. Model performance was evaluated based on Spearman rank correlation (*ρ*) and root-mean-square error (RMSE).

**Results:**

The PCA-LME models performed well (independent test: average *ρ* = 0.668, RMSE = 0.892, *P <* .001) in the prediction of the reference measurements based on in vitro data. The performance on ex vivo arthroscopic data was poorer but improved substantially after outlier exclusion (independent test: average *ρ* = 0.462 to 0.614, RMSE = 1.078 to 0.950, *P =* .019 to .008).

**Conclusions:**

NIRS is capable of nondestructive evaluation of cartilage integrity (i.e., histologic scores and proteoglycan content) under similar conditions as in clinical arthroscopy.

**Clinical Relevance:**

There are clear clinical benefits to the accurate assessment of cartilage lesions in arthroscopy. Visual grading is the current standard of care. However, optical techniques, such as NIRS, may provide a more objective assessment of cartilage damage.

Current diagnostic measures of musculoskeletal disorders involve clinical examination and imaging, e.g., x-ray imaging and magnetic resonance imaging,^1^ which lack sensitivity to pinpoint localized defects and their severity (structural damage).^2^ Conditions and injuries requiring medical interventions (e.g., a debridement, or cartilage repair) are treated in minimally invasive arthroscopy, often revealing previously unobserved focal cartilage defects.^3^ As current arthroscopic measures of evaluation are qualitative and subjective (poor interobserver reliability),^4^ a quantitative arthroscopic tool could be beneficial for the objective evaluation of chondral lesions.

Histology is the gold-standard measure of cartilage degeneration that is used to assess structural and compositional changes within the tissue.^5^ The most commonly used histologic grading systems are the modified Mankin score (0-13),^6^ Osteoarthritis Research Society International (OARSI) grading (0-6),^5^ and International Cartilage Repair Society (ICRS) grading (0-4).^7^ However, the practical clinical value of histology is limited due to invasive tissue extraction, which can greatly jeopardize cartilage integrity. Therefore, a nondestructive technique capable of providing an evaluation similar to histology would be of great value.

Optical techniques, such as near-infrared spectroscopy (NIRS)^8^ and optical coherence tomography,^9^ are capable of beyond-surface evaluation, making these techniques potentially superior to the current standard of visual evaluation during arthroscopy. The techniques use the nonionizing region of light (i.e., no deleterious effects) and the measurement can be performed in seconds. Previously, NIRS has been successfully applied for tissue diagnostics in the laboratory environment^10^, ^11^, ^12^, ^13^ and more recently in a few in vivo applications^14^^,^^15^ by using custom sterilizable probes similar to the traditional arthroscopic hook. Studies also have evaluated the histologic properties of cartilage via NIRS with moderate-to-strong correlations, especially the Mankin score, which is the most common reference for tissue integrity in animals (bovine^16^ and ovine^17^) and humans^16^^,^^18^^,^^19^ in the laboratory environment. In addition, several NIRS studies have focused on the estimation of cartilage composition, especially proteoglycan (PG) content.^8^^,^^12^^,^^20^^,^^21^

The clinical in situ application of NIRS requires a pretrained multivariate model (e.g., chemometrics and neural networks), arising from the overlapping nature of spectral peaks in the NIR range, developed using a library of both spectral and reference measurements.^22^ Lately, convolutional neural networks (CNNs) have outperformed classical chemometrics methods,^23^ such as principal component regression. These approaches, however, are yet to account for the inherent dependencies within the data that can arise, for example, from repeated measures from the same subject^13^^,^^24^—especially with valuable human data. Linear mixed-effects (LME) modeling can resolve this dependency problem and is often paired with a dimensionality reduction technique, such as principal component analysis (PCA),^25^ due to multicollinearity (i.e., a high number of collinear variables in spectral data).

In addition to the impactful role of the modeling approach, spectral preprocessing has a substantial impact on the signal interpretation and the accuracy of the resulting model.^26^ Currently, the optimal preprocessing pipelines are based on an expert opinion and, to some extent, trial-and-error. To ease the decision on optimal pipeline, open-source preprocessing pipelines, such as nippy,^27^ have become available to explore vast combinations of different preprocessing operators.

The purpose of this study was to develop the means to estimate cartilage histologic grades and PG content in ex vivo arthroscopy using NIRS. We hypothesized that NIRS would estimate cartilage lesion severity and PG content during an ex vivo arthroscopy.

## Methods

In this experimental study, NIR spectra were collected in both ex vivo arthroscopy and in vitro, and the extracted samples were subjected to extensive reference measurements. Ex vivo spectral measurements from several standardized locations (n = 19 [9 in the femur, 8 in the tibia, and 2 in the patella]) were recorded from both knees of human cadavers (N = 9, age = 68.4 ± 7.5 years) by an experienced orthopaedic surgeon (no living cartilage was assessed).^13^ The inclusion criteria for the donors were that they were scheduled for a medical obduction, had no history of knee surgery (also visually verified), and had no infectious risks. The examinations and sample extraction of this study were performed before the medical obduction and post-haste after postmortem (max 4 days) during which time the donors were stored at 4 to 7°C. In the arthroscopies, the inferior extremity of the cadaver was freely movable on a straight table and stabilized with a lateral post on the femur to allow valgisating or varisating forces created by the surgeon, to apply the setting of normal knee arthroscopy. Anteromedial and anterolateral 1 cm parapatellar interchangeable portals were created for the conventional arthroscope (4 mm, 30° inclination; Karl Storz GmbH & Co, Tuttlingen, Germany) and the novel NIRS probe, respectively. The knee joint was filled with saline using a hand pump. If necessary, an arthroscopic shaver was used to flush the intra-articular space and resect the liposynovia, thereby clearing the visualization of the areas under examination. The NIRS probe was aligned perpendicular and in contact with the cartilage under the examination based on the visualization of the conventional arthroscope. After the measurements, the condyles of the tibia and femur, and patella were harvested, followed by the extraction of cylindrical osteochondral plugs (diameter = 8 mm, the total number of extracted plugs = 303 after exclusion of 39 plugs due to completely eroded cartilage) with a drill punch machine. The plugs were subjected to in vitro spectral measurements and histologic evaluation. The local research ethics committee (decision number 134/2015, Research Ethics Committee of the Northern Savo Hospital District, Kuopio University Hospital, Kuopio, Finland) approved the study. The followed procedures were by the ethical standards of the responsible committee on human experimentation (institutional and national) and with the Helsinki Declaration of 1975, as revised in 2000.

## Near-Infrared Spectroscopy

**Fig 1:**
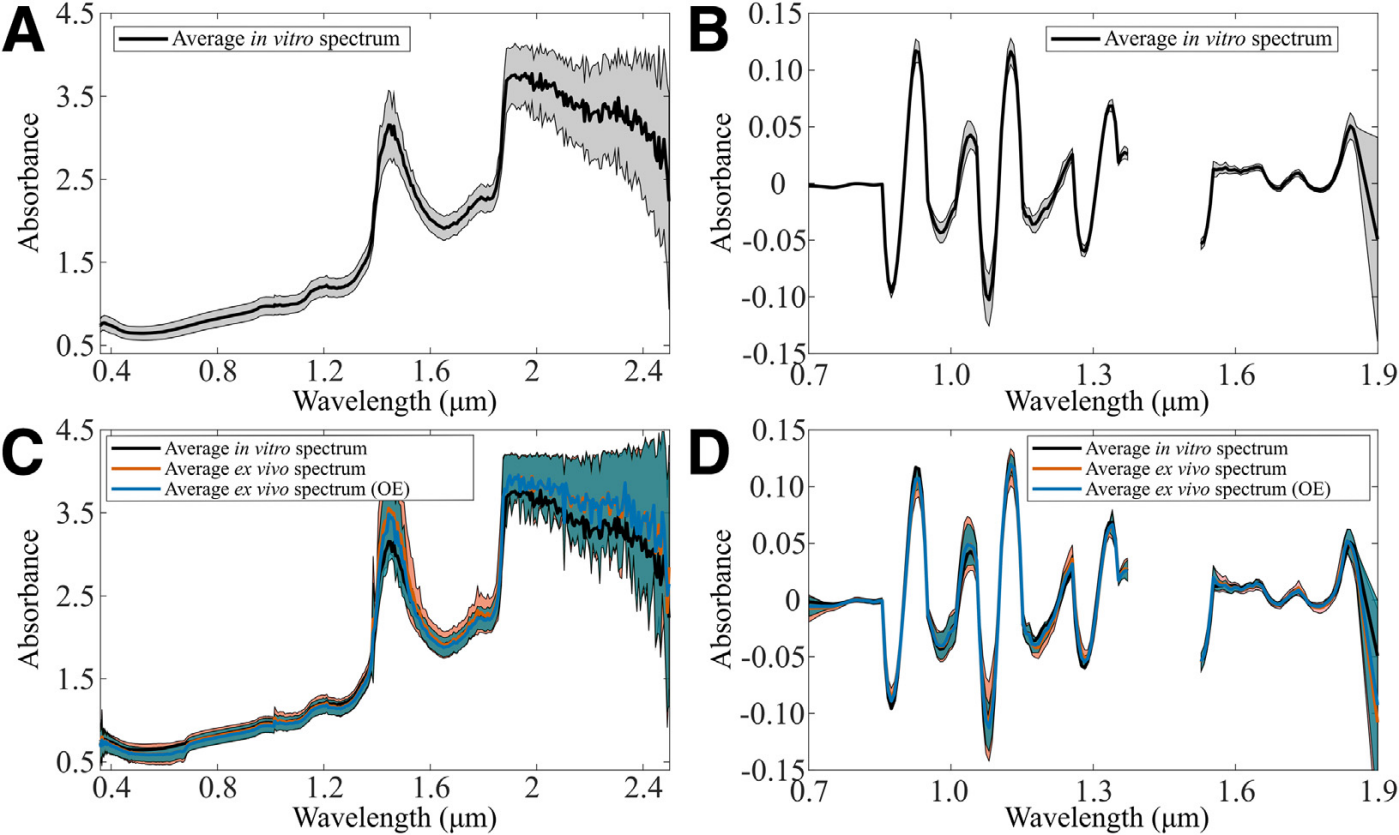
The average (lines) and standard deviation (shaded background) for nonpreprocessed spectra of in vitro (A, C) and ex vivo measurements (C) before and after outlier exclusion (OE). Similarly, preprocessed versions of the spectra are presented (B and D, the optimal preprocessing when predicting ICRS and Mankin score).

The spectra were collected both ex vivo and in vitro (Fig 1) with the hardware, consisting of 2 spectrometers (AvaSpec-ULS2048L, λ = 0.35-1.1 μm, Δλ = 0.6 nm and AvaSpec-NIR256-2.5-HSC, λ = 1.0-2.5 μm, Δλ = 6.4 nm; Avantes BV, Apeldoorn, Netherlands), a light source (AvaLight-HAL-(S)-Mini, λ = 0.36-2.5 μm, Avantes BV), and an arthroscopic optical probe.^28^ The custom-made probe resembles the conventional arthroscopic hook and has a total of 114 optical fibers (fiber diameter = 100 μm) within the sterilizable stainless-steel housing (outer diameter = 3.25 mm). Thinner fibers (with a smaller minimum bend radius) were used to support the hook-based design.

During the ex vivo spectral measurements, joint cavities were irrigated and distended with saline similarly to routine clinical arthroscopy to enhance the visibility of the articulating surfaces and to ease probe alignment. The probe was aligned perpendicular and in contact with cartilage surface under the guidance of a conventional endoscope (4 mm, 30° inclination, Karl Storz GmbH & Co.) to prevent spectral saturation from the fluid environment (effective absorber of NIR light). A total of 15 spectra were recorded per location with each spectrum consisting of 10 coadded spectra (acquisition time per location = 2.4 seconds).

The spectral measurements were repeated in vitro on osteochondral plugs extracted from the same ex vivo measurement locations. Contrary to the ex vivo measurements where optimal probe alignment could not be always ensured, in in vitro measurements the sample plugs were fixed into a goniometer (#55-841; Edmund Optics Inc., Barrington, NJ) to achieve reliable contact between the probe and the sample surface.

### Spectral Preprocessing

Before preprocessing, data from the spectral region of 0.35 to 1.10 μm were downsampled to the same resolution as data from the spectral region of 1.0 to 2.5 μm and combined. An open-source preprocessing module nippy^27^ was used in Python 3.7 to create datasets with different combinations of preprocessing as highlighted by Torniainen et al. The preprocessing options used included smoothing, scatter correction techniques, and trimming. A third-degree Savitzky-Golay filter with 0th (i.e., smoothing), first, and second derivatives with different filter windows (5-47) were evaluated. For scatter correction, standard normal variate and localized standard normal variate (LSNV, windows = 2*i*, *i* = 1-6) were tested. The spectral regions of 0.70 to 1.90 μm, 0.75 to 1.85 μm, 0.70 to 1.375 μm and 1.525 to 1.90 μm; 0.75 to 1.375 μm and 1.525 to 1.85 μm were tested separately. The visible spectral region (0.35-0.70 μm) was excluded due to the interference originating from the endoscope. The spectral region 1.90 to 2.50 μm was excluded due to the poor signal-to-noise ratio (high water absorption).

### Histology

**Fig 2:**
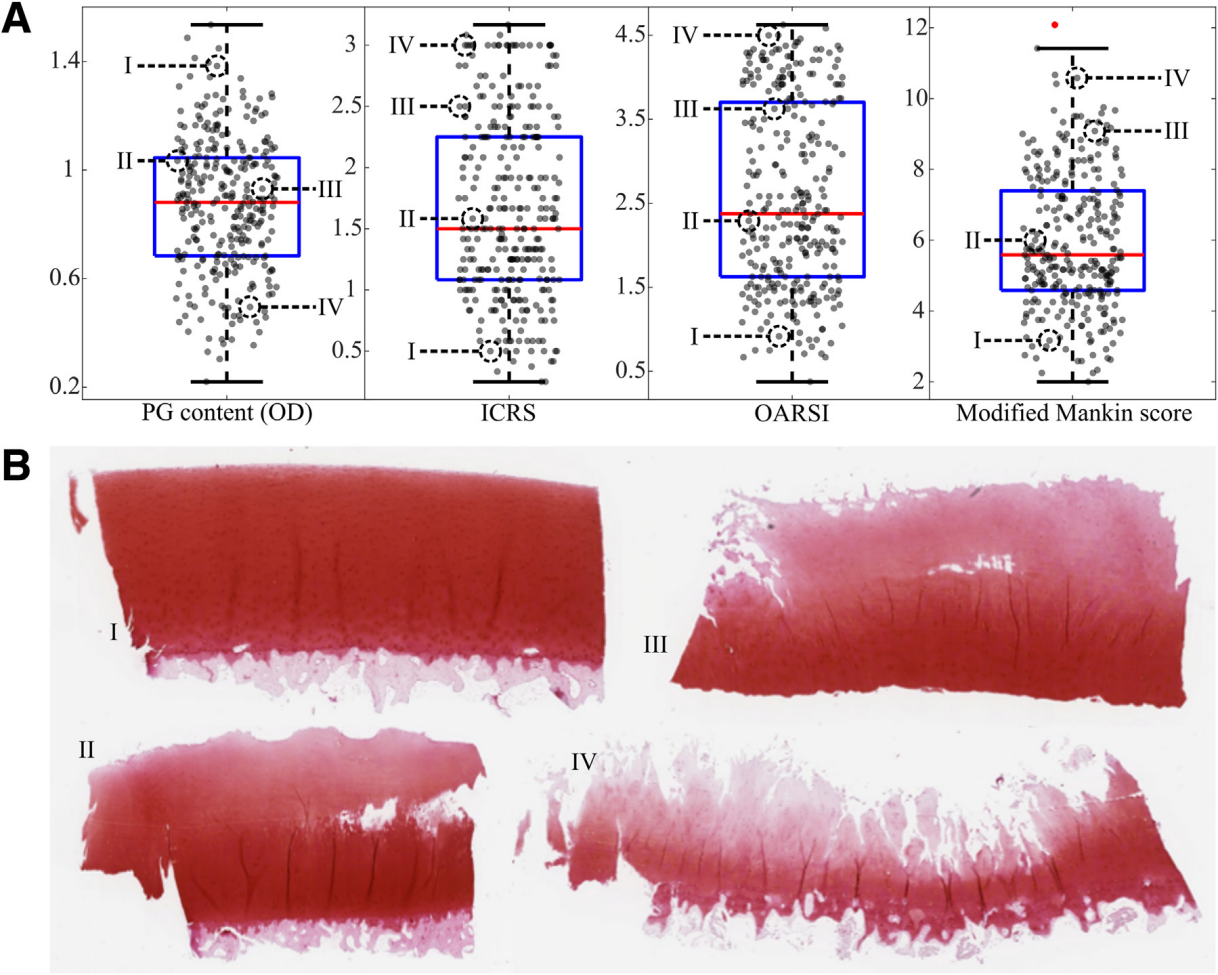
Boxplots along with individual samples (gray) and outliers (red) of reference variables (A). Four representative microscopy images (B, I-IV) are presented with their reference parameter values indicated in subfigure A.

The osteochondral plugs were halved and one half was decalcified in ethylenediaminetetraacetic acid, cut into 3-μm thick sections (n = 3), and stained with Safranin-O (attracted by PGs). Digital densitometry system, consisting of a light microscope (Nikon Microphot-FXA; Nikon Co., Tokyo, Japan) with a monochromatic light source (wavelength 492 ± 8 nm) and a 12-bit CCD camera (ORCA-ER; Hamamatsu Photonics K.K., Hamamatsu, Japan), was used to determine sections optical density (OD ∼ PG content). The system was calibrated with neutral density filters (0-3.0). The severity of OA was evaluated with 3 histologic grading systems: modified Mankin score,^6^ OARSI grading,^5^ and ICRS grading^7^ (Fig 2). Four independent assessors (M.P., R.S., M.H., and N.H.) scored the sections in a randomized order and the final score for each sample was determined as the average over the 3 sections. The same sections were used for digital densitometry and histologic grading.

### Regression Analysis

Before regression analysis, spectral (multivariate) and reference parameter (univariate) outliers were investigated. The in vitro spectra were visualized to ensure the absence of hardware-related recording errors. In the preprocessing of ex vivo spectra, 10 hardware-related outliers were identified and excluded. For univariate values, the normality of distribution (non-normal for all references) was determined with the one-sample Kolmogorov–Smirnov test and, thus, any values exceeding 3 median absolute deviations from the reference median were excluded.

PCA was used due to its ability to reduce the high dimensionality and collinearity of datasets, thereby enabling less computationally exhaustive modeling and outlier estimation, as well as reducing the chances of overfitting. PCA scores along with the nested data (i.e., patient, left/right, femur/tibia/patella, and measurement site) were used as inputs for the LME model.

In the modeling, 8 cadavers were assigned as the training set and a single cadaver was assigned as the independent test set. The test set was subsequently changed (9 iterations) until all cadavers were used (also known as nested cross-validation). The in vitro models were calibrated and optimized using 10-fold cross-validation with the number of PCA scores limited to 12. The model with the smallest root-mean-square error of cross-validation (RMSECV) was selected to minimize overfitting. The optimal preprocessing pipeline was selected based on the highest median Spearman rank correlation in the independent test set.

### Arthroscopic Outlier Detection

Due to the relatively narrow joint cavities and limited field of view in the ex vivo measurements, optimal contact between the cartilage surface and probe could not always be ensured. Furthermore, the high water content of cartilage makes the spectral separation between the measurements with good and bad contact especially challenging and, thus, a classifier was trained to identify spectra with non-optimal contact. The performance of classifiers, including fine k-nearest neighbors (kNN), weighted kNN, and support vector machines (SVM), was investigated due to their superior performance in the initial testing. The classifier optimization was performed as follows: the cross-validated PCA-LME model was used to predict the properties based on the ex vivo spectra of the 8 cadavers (same as in training). If the error between the predicted and reference value was greater than a set threshold (2 × RMSECV, 3 × RMSECV, or half set as outliers), the label was set to 1 (= outlier), otherwise to 0. These labels along with PCA scores (N = 12) of ex vivo spectra were then used to train a 10-fold cross-validated classifier, which was used to classify the remaining independent arthroscopic measurements (one cadaver). The effect of preprocessing pipeline on classifier performance was also investigated. Ultimately, the performance of the same retained locations in both in vitro and ex vivo should be equal—this was used as an indicator (along with classifier accuracy and F1-score) to determine the optimal combination of algorithm, threshold, and preprocessing. The combinations that classified <10% or >90% of ex vivo spectra as outliers were not included as these were not considered realistic.

### Statistical Analysis

The PCA-LME model performance was evaluated based on performance in calibration (Spearman rank correlation [*ρ*], RMSECV) and the independent test (*ρ*, RMSE). As a statistic, the median was chosen over the average as it is less susceptible to outliers. Model and classifier training were performed in MATLAB (R2020b, MathWorks, Natick, MA). The level of significance was set at *P <* .05. Data of the current study are available from the corresponding author on reasonable request.

## Results

The distributions of reference properties were all non-normal with a single outlier detected with the Mankin score (Fig 2). The optimal PCA-LME models had a moderate performance with the histology scores (i.e., ICRS, OARSI, and Mankin) and slightly poorer performance with PG content (Table 1: PCA-LME). The optimal preprocessing pipelines always included scatter correction (i.e., LSNV); furthermore, for the histologic scores, the combination of spectral regions of 0.70 to 1.375 and 1.525 to 1.90 μm was optimal (Table 2). The optimal preprocessing pipelines for ICRS and Mankin scores were identical.

**Table 1:** Performance Metrics as Median (Interquartile Range) for Optimized PCA-LME Model, Classifier, and Ex Vivo Predictions

				PG Content	ICRS	OARSI	Mankin
In vitro	PCA-LME	Train	*ρ*	0.739 (0.033)	0.757 (0.012)	0.759 (0.009)	0.734 (0.012)
			RMSECV	0.185 (0.005)	0.536 (0.014)	0.800 (0.032)	1.381 (0.043)
			N	8 (2)	8 (2)	7 (1.25)	8 (1.25)
		Test	*ρ*	0.583 (0.371)	0.687 (0.253)	0.731 (0.291)	0.669 (0.258)
			RMSE	0.256 (0.148)	0.628 (0.206)	0.911 (0.282)	1.772 (0.607)
		SRL	*ρ*	0.561 (0.420)	0.715 (0.387)	0.671 (0.263)	0.693 (0.252)
			RMSE	0.246 (0.147)	0.602 (0.150)	0.830 (0.368)	1.718 (0.641)
Ex vivo	All	Test	*ρ*	0.421 (0.505)	0.492 (0.274)	0.555 (0.230)	0.379 (0.253)
			RMSE	0.286 (0.173)	0.825 (0.340)	1.057 (0.201)	2.145 (0.729)
	Classifier	Train	Accuracy	0.775 (0.027)	0.850 (0.010)	0.850 (0.015)	0.807 (0.009)
			F1-score	0.775 (0.027)	0.859 (0.010)	0.849 (0.014)	0.710 (0.020)
		Test	Accuracy	0.484 (0.042)	0.581 (0.125)	0.538 (0.050)	0.687 (0.110)
			F1-score	0.431 (0.082)	0.533 (0.138)	0.507 (0.021)	0.503 (0.045)
			OutlierS %	52.7 (9.0)	61.1 (21.6)	41.9 (8.7)	78.9 (4.1)
			OutlierN %	0 (4.4)	24 (19.6)	0 (15.8)	24 (13.9)
			*ρ*	0.522 (0.481)	0.716 (0.315)	0.630 (0.227)	0.586 (0.383)
			RMSE	0.274 (0.173)	0.703 (0.247)	0.946 (0.193)	1.876 (0.548)
		SRL	*ρ*	0.513 (0.481)	0.686 (0.305)	0.599 (0.210)	0.515 (0.368)
			RMSE	0.274 (0.165)	0.703 (0.256)	0.952 (0.171)	1.876 (0.548)
							

Same locations (SRL) are presented to enable better comparison between in vitro and ex vivo performance. OutlierS presents the percentage of spectra excluded by the classifier and OutlierN the percentage of excluded measurement locations (i.e., the location was excluded if all 15 spectra were outliers).

ICRS, International Cartilage Repair Society; OARSI, Osteoarthritis Research Society International; PCA-LME, principal component analysis–linear mixed-effects; PG, proteoglycan; RMSECV, root mean square error of cross-validation.

**Table 2:** Optimal Preprocessing Pipelines for Different Models With Their Derivative Order (deriv_order) and Window Size (filter_win)

		Scatter Correction	Preprocessing	Spectral Range, μm
PCA-LME	PG content	LSNV_win: 10	deriv_order: 1, filter_win: 23	0.70-1.90
	ICRS	LSNV_win: 6	deriv_order: 2, filter_win: 15	0.70-1.375, 1.525-1.90
	OARSI	LSNV_win: 2	deriv_order: 0, filter_win: 47	0.70-1.375, 1.525-1.90
	Mankin	LSNV_win: 6	deriv_order: 2, filter_win: 15	0.70-1.375, 1.525-1.90
Classifier	PG content	LSNV_win: 6	deriv_order: 0, filter_win: 35	0.70-1.375, 1.525-1.90
	ICRS	LSNV_win: 2	deriv_order: 0, filter_win: 7	0.75-1.375, 1.525-1.85
	OARSI	LSNV_win: 4	deriv_order: 0, filter_win: 11	0.75-1.375, 1.525-1.85
	Mankin	LSNV_win: 6	deriv_order: 0, filter_win: 31	0.70-1.90

ICRS, International Cartilage Repair Society; LSNV, localized standard normal variate; OARSI, Osteoarthritis Research Society International; PCA-LME, principal component analysis–linear mixed-effects; PG, proteoglycan.

**Fig 3:**
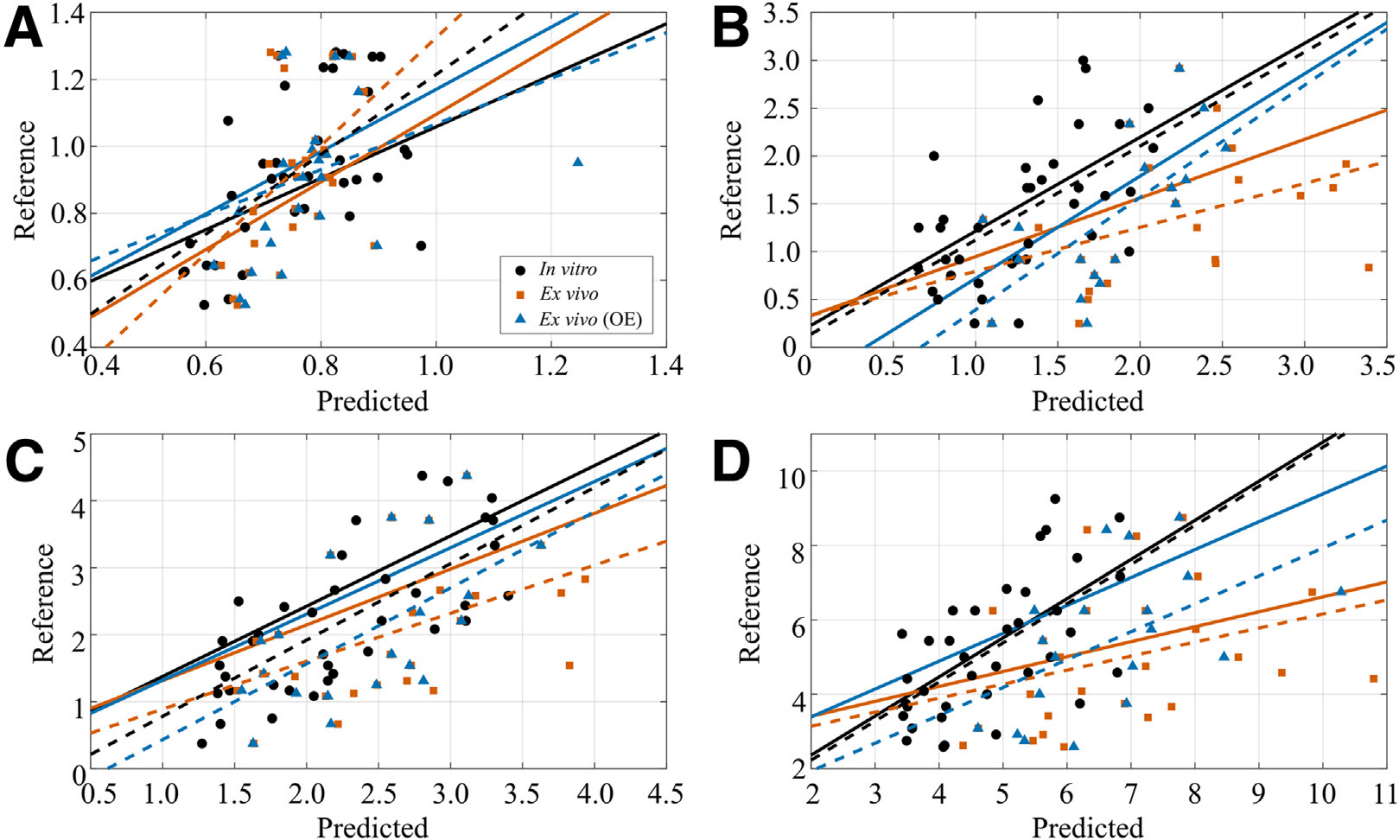
Predicted and reference values for PG content (A), ICRS (B), OARSI (C), and modified Mankin (D) scores are presented for the independent test (1 iteration out of 9), including their linear fits (dashed lines) and the median linear fit of the 9 iterations (solid lines). In vitro, ex vivo, and ex vivo after outlier exclusion (OE) are presented separately. (ICRS, International Cartilage Repair Society; OARSI, Osteoarthritis Research Society International; PG, proteoglycan.)

Before outlier classification, the initial performance of the PCA-LME models on ex vivo spectra was assessed (Table 1: All Test). The optimal combination of preprocessing, classifier algorithm, and threshold substantially improved model performance on the ex vivo performance of the independent test set (*ρ* = 0.462 to 0.614, RMSE = 1.078 to 0.950, *P =* .019 to .008, Fig 3). To better compare in vitro and ex vivo performance in the test set, a comparison of same retained locations revealed slightly different performance (*ρ =* 0.660, RMSE = 0.849, *P =* .001 vs *ρ =* 0.578, RMSE = 0.951, *P =* .018, respectively). In addition, to estimate model reliability, the prediction error was assessed in 4 classes (i.e., dividing reference ranges to four equally spaced subranges), which revealed the prediction error to be smallest with the 2 middle classes. Although the percentage of outlier spectra (Table 1: OutlierS) was relatively high, the exclusion percentage of measurement locations was substantially lower (Table 1: OutlierN). The variability in the percentage of outliers between reference properties relates to the differences in spectral preprocessing, the performance of the PCA-LME model (RMSECV as a metric for data labeling), and the accuracy of the classifier (prediction reliability).

The optimal preprocessing pipelines for classification systematically included scatter correction (i.e., LSNV) and smoothing (i.e., no derivative preprocessing, Table 2). The outlier thresholds of 3 × RMSECV and 50% were optimal for the histology scores and PG content, respectively. The optimal classifiers for PG content, ICRS, OARSI, and modified Mankin score were fine-kNN, SVM, weighted kNN, and fine-kNN, respectively. Overall, none of the algorithms performed systematically better than the others and SVM classified more spectra as outliers than the kNN algorithms. We also investigated classifier performance when using the same preprocessing pipeline as in the modelling; however, the ex vivo performance was systematically worse compared with the optimized pipeline (*ρ* = 0.452 to 0.614, RMSE = 1.032 to 0.950, *P* = .027 to .008).

Outlier exclusion decreased the standard deviation of ex vivo spectra by 23.3% and also decreased the absorption at 1.4 μm (water peak, Fig 1), depicting the exclusion of spectra with saline interference (i.e., more water). Most importantly, the visualization of *ex vivo* spectra before and after outlier exclusion confirmed that extreme spectra were excluded.

## Discussion

In this study, we demonstrate that NIRS is capable of estimating cartilage lesion severity and PG content during an ex vivo arthroscopy, thus validating the hypothesis. The in vitro performance of PCA-LME models was moderate to strong and the ex vivo arthroscopic performance was slightly poorer, which was nevertheless substantially improved by excluding outlying spectra. We therefore, believe the technique could provide previously unobtainable diagnostic information during arthroscopic surgery.

NIRS has been previously applied to estimate cartilage histologic scores in animals^29^ and humans^16^^,^^18^^,^^19^ in vitro. In addition, ICRS scores based on visual evaluation in arthroscopy have been associated with in vitro^19^^,^^30^ and in vivo^31^ NIRS measurements. The human studies^16^^,^^18^^,^^19^^,^^30^^,^^31^ have employed varying spectral ranges and analysis techniques with none accounting for the spatial dependency caused by multiple measurements per subject or validating model performance by independent testing. McGoverin et al.^16^ reported a similar cross-validation error (PLS: calibration R^2^ = 0.84, RMSECV = 1.3) on modified Mankin score with the spectral region 1.11-2.27 μm, whereas Afara et al.^18^ presented slightly poorer performance (PLS: calibration R^2^ = 0.83, RMSECV = 1.6) on Mankin score using the spectral region of 0.40-1.10 μm. In addition, Stumpfe et al.^19^ demonstrated moderate correlation (PLS: *ρ* = 0.54) between the spectral region 0.95 to 1.70 μm and Mankin score but no error statistics or any means of cross-validation were reported. Marticke et al.^30^ reported a negative relationship (*ρ* = –0.47) between arthroscopic ICRS score and a spectral characteristic value (estimated as a linear combination of 2 spectral peaks), whereas Spahn et al.^31^ reported a positive association with spectral ratio (ratio of the same 2 peaks). Direct comparison of the aforementioned studies is limited due to their simplistic analysis approach. Overall, the findings of this study agree with in vitro performance of previous studies and extend technique validity and application for in vivo application.

Previous studies associating PG content with NIR spectra have focused on bovine^20^^,^^21^ and equine cartilage.^8^^,^^12^ Few studies have also investigated the PG content of engineered cartilage constructs.^32^, ^33^, ^34^ The bovine studies of Brown et al.^20^ and Afara et al.^21^ both used the spectral region 0.8 to 2.5 μm, PLS regression, and assessed PG content after various stages of artificial depletion. Brown et al.^20^ presented a significant distinction between normal and PG depleted samples, whereas Afara et al.^21^ demonstrated a superior cross-validated performance (calibration R^2^ = 93.76, RMSECV = 0.573) based on data within the spectral regions 0.8 to 1.0 μm and 1.55 to 1.84 μm. The calibration correlation of Afara et al.^21^ is substantially greater than that found in this study (*ρ* = 0.739, RMSECV = 0.185), whereas the cross-validated errors are similar, considering the ranges of OD values (0.2-1.5) and PG scores (0-4) in this and their study,^21^ respectively. The higher correlation could be explained by their use of leave-one-out (vs k-fold) cross-validation and smaller sample size. Sarin et al.^8^ applied a similar cross-validation scheme and also validated the performance for arthroscopy on equine. The validation performance of their CNN (*ρ* = 0.691, RMSECV = 0.274) was fairly similar. Furthermore, the validation performance on arthroscopic spectra was inferior to the aforementioned performance, similar to the present study. Most importantly, it’s evident that prediction of PG content is possible and reproducible both for equine and human cartilage; although, their thickness varies greatly (0.14-1.36 mm and 1.23-5.90 mm, respectively^8^^,^^13^). Due to the superior performance of sophisticated machine-learning approaches over conventional chemometrics techniques,^35^ the combination of CNN and protocols accounting for data dependency could benefit future studies.

The NIRS literature of the aforementioned publications provides the groundwork for clinical adaptation of NIRS in cartilage assessment. Nowadays, arthroscopic cartilage evaluation usually relies on visual estimation and instrument palpation, which has limited reliability in cartilage defect grading.^4^^,^^36^ Here, NIRS enables the estimation of PG content and defect severity in a situation resembling that of arthroscopic surgery without the need for destructive sample extraction. This nondestructive evaluation could enable the objective classification of cartilage defects and further enable the monitoring of potential treatment options in cartilage repair. In addition, the technique enables the mapping of the extent of traumatic cartilage injuries that would assist in the treatment decision-making and open new possibilities to evaluate the prognosis of a cartilage defect in joint trauma. For example, post-traumatic cartilage degeneration is a well-known consequence of an anterior cruciate ligament rupture, even after a successful anterior cruciate ligament reconstruction.^37^^,^^38^ NIRS could enable the evaluation of this post-traumatic process in the knee and other joints.

Outlier detection is essential in both the initial model training and validation, followed by ensuring the validity of new data (i.e., no unreliable predictions). For this study, median-based statistics were chosen due to their inherent property of being less sensitive to outliers (compared with average). Estimation of spectral (multivariate) outliers is challenging and, thus, dimension reduction techniques, such as PCA, have been popular.^8^^,^^13^^,^^15^ Furthermore, due to the high water content of cartilage, the spectral separation between the measurements with poor and good contact is especially challenging and requires further validation (i.e., a designated study). Therefore, the spectral outliers highly resemble the nonoutlier spectra. In 2 studies by Sarin et al.,^8^ a 3-dimensional volume was created based on PCA scores of in vitro data and arthroscopic spectra falling outside this volume were deemed outliers. The exclusion percentages were 4.5% to 23.5% and 3.1%.^8^^,^^15^ Prakash et al.^13^ used a similar outlier exclusion as in this study with similar accuracy of 50% to 70% in the test set but a relatively lower percentage of outliers (33%). Interestingly, after outlier exclusion, their correlation coefficients improved but error variance substantially increased. In this study, both the error and its variance decreased after outlier exclusion. Several studies^14^^,^^31^^,^^39^ focusing on in vivo spectral measurements do not provide any means for outlier exclusion, although highlighting the difficulty of in vivo measurements.^39^ In future studies, outlier detection may also benefit from the latest innovations, such as extended isolation forests.^40^

### Limitations

Some limitations were evident in this study. The number of cadavers was relatively low and could lead to limited range (nonrepresentative) of reference properties; however, several locations were assessed in extensive laboratory measurements, thereby sufficiently increasing the number of observations. A relatively high percentage of outlier spectra depicts the challenge in probe alignment within the joint cavity to ensure optimal contact with the cartilage surface, as also highlighted by Spahn et al.^39^ However, the percentage of outlier locations (i.e., all 15 spectra excluded) was substantially lower, indicating that a successful measurement was acquired in most of the locations.

## Conclusions

NIRS is capable of nondestructive evaluation of cartilage integrity (i.e., histologic scores and PG content) under similar conditions as in clinical arthroscopy.

## Supplementary Data

ICMJE author disclosure forms
